# Faricimab for neovascular age-related macular degeneration and diabetic macular edema: from preclinical studies to phase 3 outcomes

**DOI:** 10.1007/s00417-024-06531-9

**Published:** 2024-06-07

**Authors:** Hansjürgen Agostini, Francis Abreu, Caroline R. Baumal, Dolly S. Chang, Karl G. Csaky, Anna M. Demetriades, Laurent Kodjikian, Jennifer I. Lim, Philippe Margaron, Jordi M. Monés, Tunde Peto, Federico Ricci, Matthias Rüth, Rishi P. Singh, Ivaylo Stoilov, Balakumar Swaminathan, Jeffrey R. Willis, Peter D. Westenskow

**Affiliations:** 1 Eye Center, Medical Center, Faculty of Medicine, University of Freiburg, Freiburg, Germany; 2 Genentech, Inc., South San Francisco, CA USA; 3 Tufts Medicine New England Eye Center, Boston, MA USA; 4 Apellis Pharmaceuticals, Waltham, MA USA; 5 Retina Foundation of the Southwest, Dallas, TX USA; 6 Department of Ophthalmology, Stanford University School of Medicine, Stanford, CA USA; 7 Department of Ophthalmology, Croix-Rousse University Hospital, Hospices Civils de Lyon, Lyon, France; 8 CNRS UMR 5510 Mateis, INSA, University of Lyon I, Villeurbanne, France; 9 Department of Ophthalmology and Visual Sciences, University of Illinois College of Medicine, University of Illinois at Chicago, Chicago, IL USA; 10 F. Hoffmann-La Roche Ltd., Basel, Switzerland; 11 Centro Médico Teknon, Institut de La Màcula and Barcelona Macula Foundation, Barcelona, Spain; 12 Centre for Public Health, Queen’s University Belfast, Belfast, UK; 13 Department of Experimental Medicine, University “Tor Vergata”, Rome, Italy; 14 Cleveland Clinic Florida, Stuart, FL USA; 15 F. Hoffmann-La Roche Ltd., Mississauga, ON Canada

**Keywords:** Angiopoietin-2, Faricimab, Retinal vascular disease, Vascular endothelial growth factor-A, Vascular stability

## Abstract

Intravitreal anti–vascular endothelial growth factor (VEGF) therapy is the standard of care for diabetic macular edema (DME) and neovascular age-related macular degeneration (nAMD); however, vision gains and anatomical improvements are not sustained over longer periods of treatment, suggesting other relevant targets may be needed to optimize treatments. Additionally, frequent intravitreal injections can prove a burden for patients and caregivers. Angiopoietin-2 (Ang-2) has been explored as an additional therapeutic target, due to the involvement of Ang-2 in DME and nAMD pathogenesis. Recent evidence supports the hypothesis that targeting both VEGF and Ang-2 may improve clinical outcomes in DME and nAMD compared with targeting VEGF alone by enhancing vascular stability, resulting in reduced macular leakage, prevention of neovascularization, and diminished inflammation. Faricimab, a novel bispecific antibody that targets VEGF-A and Ang-2, has been evaluated in clinical trials for DME (YOSEMITE/RHINE) and nAMD (TENAYA/LUCERNE). These trials evaluated faricimab against the anti-VEGFA/B and anti–placental growth factor fusion protein aflibercept, both administered by intravitreal injection. In addition to faricimab efficacy, safety, and pharmacokinetics, durability was evaluated during the trials using a treat-and-extend regimen. At 1 year, faricimab demonstrated non-inferior vision gains versus aflibercept across YOSEMITE/RHINE and TENAYA/LUCERNE. In YOSEMITE/RHINE, faricimab improved anatomic parameters versus aflibercept. Reduction of central subfield thickness (CST), and absence of both DME and intraretinal fluid were greater in faricimab- versus aflibercept-treated eyes. In TENAYA/LUCERNE, CST reductions were greater for faricimab than aflibercept at the end of the head-to-head phase (0–12 weeks), and were comparable with aflibercept at year 1, but with less frequent dosing. CST and vision gains were maintained during year 2 of both YOSEMITE/RHINE and TENAYA/LUCERNE. These findings suggest that dual Ang-2/VEGF-A pathway inhibition may result in greater disease control versus anti-VEGF alone, potentially addressing the unmet needs and reducing treatment burden, and improving real-world outcomes and compliance in retinal vascular diseases. Long-term extension studies (RHONE-X, AVONELLE-X) are ongoing. Current evidence suggests that dual inhibition with faricimab heralds the beginning of multitargeted treatment strategies inhibiting multiple, independent components of retinal pathology, with faricimab providing opportunities to reduce treatment burden and improve outcomes compared with anti-VEGF monotherapy.

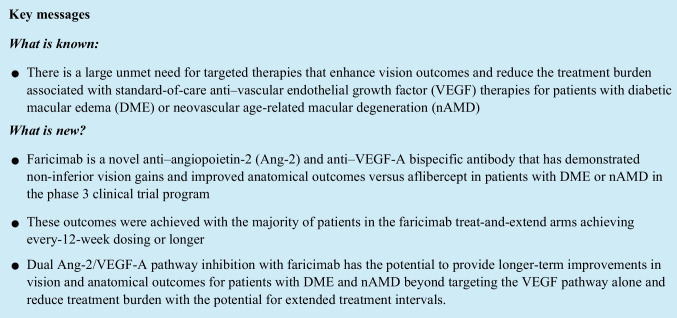

## Introduction

Intravitreal anti–vascular endothelial growth factor (VEGF) monotherapy is the standard of care for diabetic macular edema (DME) and neovascular age-related macular degeneration (nAMD) [1–3]. However, in DME, the Diabetic Retinopathy Clinical Research Network Protocol I and Protocol T comparative effectiveness studies found that after 2 years, vision stopped improving and vision gains were not sustained in large proportions of patients [4, 5]. Many patients also had persistent retinal thickening after 2 years [4, 5]. In trials of monthly ranibizumab in DME, up to 10% of eyes still progressed from early disease (i.e., non-proliferative diabetic retinopathy [NPDR]) to more severe disease (i.e., proliferative diabetic retinopathy [PDR]) after 2 years [6]. In the Protocol W trial of the anti–VEGF-A and anti–placental growth factor fusion protein aflibercept in DME, 4-year cumulative probability of developing PDR was 33.9% in the aflibercept arm versus 56.9% in the sham arm; additionally, 4-year cumulative incidence of center-involving DME with vision loss was 11.3% in the aflibercept group and 19.1% in the sham group [7]. This progression may reflect the complex and multifactorial pathophysiology of diabetic retinopathy (DR)/DME; therefore, novel treatments targeting other disease mechanisms beyond VEGF inhibition should be explored [6, 8].

In patients with nAMD, visual acuity (VA) gains observed during the first 2 years of designated anti-VEGF therapy were not maintained at 5 years in the Comparison of Age-related macular degeneration Treatment Trials follow-up study [9, 10]. Similarly, in HORIZON, an open-label extension study of the anti–VEGF-A antibody ranibizumab [11], incremental declines from initial VA gains were observed after 2 years. With aflibercept, in the VIEW studies, mean 1-year VA gains were broadly maintained but with small losses at years 2 and 4, and anatomic deterioration was reported [12, 13]. Furthermore, in the SEVEN-UP study, although approximately one-third of patients treated with ranibizumab had a good visual outcome after 7 years, one-third had a best-corrected VA (BCVA) of 20/200 or worse [14].

Analyses of real-world clinical practice data have shown that many patients do not achieve vision gains equivalent to those reported in clinical trials, and that many receive fewer anti-VEGF injections than in clinical trials [15–22]. The frequent intravitreal injections and close monitoring required to achieve optimal outcomes with intravitreal anti-VEGF monotherapy are a burden for patients and their caregivers [16, 20], and several risk factors for suboptimal treatment outcomes have been reported, including poor baseline VA, sociodemographic factors, older age, and co-existing health issues [18, 23, 24]. DME and nAMD have a multifactorial pathogenesis, including the influence of inflammatory mediators and growth factors, meaning that VEGF inhibition alone cannot address all aspects of each disease [8, 25].

As such, there remains a significant opportunity for improvement in treatment outcomes for patients with retinal vascular diseases, and a need for additional therapeutic strategies to reduce treatment burden and optimize vision outcomes for patients with DME and nAMD.

This review examines the rationale for targeting two pathways, angiopoietin-2 (Ang-2) in addition to VEGF, for treatment of DME and nAMD. We assess the results from recent clinical trials in patients with DME and nAMD that evaluated the efficacy, safety, pharmacokinetics, and durability of dual VEGF-A and Ang-2 inhibition with faricimab, a novel bispecific antibody directed against these two molecules.

## The Ang/Tie pathway in vascular stability and remodeling

**Fig. 1:**
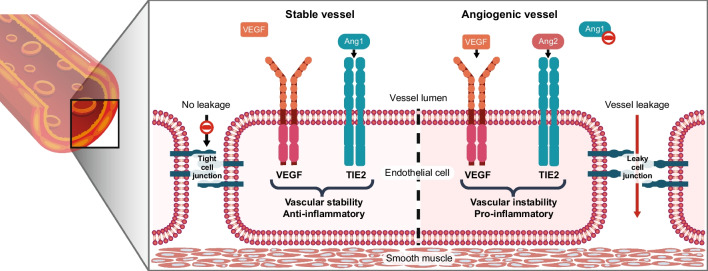
Simplified overview of the contribution of Ang/Tie2 and VEGF signaling to the regulation of vascular homeostasis. During the switch from stable vasculature to angiogenesis, Ang-1 levels are unchanged, but Ang-2 production increases and Ang-2 outcompetes Ang-1 for binding to Tie2. *Ang-1* angiopoietin-1, *Ang-2* angiopoietin-2, *Tie2* tyrosine kinase with immunoglobulin-like and epidermal growth factor homology domains-2, *VEGF* vascular endothelial growth factor

The Ang/tyrosine kinase with immunoglobulin-like and epidermal growth factor homology domains (Tie) pathway is essential for regulating vascular stability, angiogenesis, vascular permeability, and inflammation (Fig. 1) [26–28]. In healthy adult retinas with quiescent blood vessels, angiopoietin-1 (Ang-1) binds to and activates the Tie2 receptor, promoting vascular stability by stimulating endothelial cell survival and endothelial cell junction stability [26, 27]. In disease states, hypoxia and inflammation trigger an angiogenic switch that enhances production of Ang-2, which outcompetes (overwhelms) Ang-1 for Tie2 receptor occupancy [26, 27]. This sequence of events induces vascular permeability and instability through pericyte drop-out, leukocyte recruitment and adhesion, and weakening of endothelial cell junctions [27].

Tie2 is a tyrosine kinase receptor expressed primarily on the cell surface of endothelial cells and some hematopoietic cells [27, 29–31], and it activates the phosphatidylinositol 3-kinase-protein kinase B pathway [27, 29–31]. Ang-2 is expressed by endothelial and extravascular cells, and is stored in intracellular Weibel-Palade bodies, where it is generally present at low levels except for in the microvascular endothelium [32] (the endothelium most affected in DR and AMD). Ang-2 is localized to active sites of angiogenesis/vascular remodeling and is released from Weibel-Palade bodies in response to thrombin or histamine [32, 33]. The role of Ang-2 in angiogenesis and vascular remodeling is further supported by studies of manipulation of Ang-2 expression in mouse models [34–36]. Although Ang-2 is not required for retinal development in the embryo, it is needed shortly after birth [37, 38]. Studies in animal models of disease have shown that Ang-2 collaborates with VEGF, alongside VEGF-independent effects, to activate pathological angiogenesis and promote vascular instability [39].

## Vascular instability, VEGF, and Ang-2 in DME and DR

DME and DR are multifactorial in origin; microangiopathy and neurodegeneration, together with the inter-related effects of chronic hyperglycemia, hypoxia, low-grade inflammation, and proinflammatory cytokine release, trigger a number of changes, including pericyte loss, endothelial cell loss, capillary basal lamina thickening, retinal ischemia, and the breakdown of the blood-retina barrier [40–42]. These changes lead to the formation of microaneurysms, intraretinal microvascular abnormalities that are characteristic of NPDR, and to retinal neovascularization and vitreous hemorrhages, which are indicative of PDR [2, 43, 44]. Furthermore, as a result of the blood-retina barrier breakdown, localized fluid imbalances can lead to macular edema and thickening of the central fovea characteristic of center-involving DME [2, 43, 45–47].

Several processes that are central to the pathogenesis of DME, such as vessel instability through pericyte loss, loss of endothelial tight junctions, increased vascular permeability, and ischemia, can be experimentally manipulated in animal models, and are influenced by Ang-2 activity [27, 43, 47–49]. These features of DME pathogenesis are also associated with disease states, including inflammation, obesity, infectious disease (including complications such as sepsis), cancer, and cardiovascular disease. Ang-2 activity has also been shown to be involved in vascular instability in these conditions [50].

In a mouse ischemic retinopathy model, Ang-2 expression was increased in retinal endothelial cells and was necessary for retinal neovascularization [37, 51]. A 30-fold upregulation of Ang-2 was observed in the retinas of diabetic rats prior to the onset of pericyte loss, and intravitreal injection of Ang-2 induced dose-dependent pericyte loss without affecting vessel formation in normal rats [52]. In the same report, heterozygous Ang-2 deficiency in mice prevented diabetes-induced pericyte loss and reduced the number of acellular capillary segments [52]. Furthermore, inhibiting the activity of vascular endothelial protein tyrosine phosphate (VE-PTP), a negative regulator of Tie2, has been shown to reduce ischemia-induced retinal neovascularization in mice [53]. These findings suggest that Ang-2/Tie2 plays a role in retinal neovascularization in the ischemic retina, and that inhibition of Ang-2 could be an effective approach to reduce angiogenesis in ischemic retinal diseases.

In addition to animal model data, findings in patients with ischemic retinal disease also support a role for Ang-2/Tie2 and VEGF pathways in retinal neovascularization. In patients with DR and retinal vein occlusion, vitrectomized epiretinal membranes showed upregulation of Ang-2, Tie2, and VEGF, but not Ang-1, proteins [54]. Additionally, intraocular VEGF is upregulated in the vitreous of patients with PDR and DME [55–57]. Other studies have corroborated increased Ang-2 and VEGF in the vitreous of patients with DR, as well as found increased concentrations of matrix metalloproteases-2 and -9, erythropoietin, and transforming growth factor-β [58, 59]. However, while VEGF-induced vascular leakage plays a key role in DME, it is not the whole story. For instance, alterations in the intraocular cytokine milieu after anti-VEGF therapy (with bevacizumab) suggest the presence of compensatory mechanisms involving other cytokines and growth factors in response to VEGF inhibition [60]. Connective tissue growth factor plays a role in DR, suggesting that treatments targeting the fibrotic pathway may be required in DR [61]. Macular edema resulting from vascular leakage can cause irreversible vision loss; patients may therefore benefit from treatment that targets early vascular destabilization and remodeling in NPDR, before progression to PDR and/or DME [62]. In summary, findings from preclinical studies and from patients with DR and DME suggest that Ang-2 is a potential target for evaluation in this context due to its influence on vascular stability, independent from VEGF.

## Vascular instability, VEGF, and Ang-2 in nAMD

In nAMD, pathological changes occur within the choroid-retinal pigment epithelium (RPE) complex. Thickening and disruption of Bruch’s membrane and the presence of sub-RPE deposits and drusen contribute to oxidative stress in the RPE due to N-retinylidene-N-retinylethanolamine (A2E) accumulation, and chronic inflammation via the complement cascade and membrane attack complex, thereby causing loss of the choriocapillaris [63–66]. Disruption of the cross-talk between the RPE and choriocapillaris endothelial cells results in choriocapillaris loss [63–68]. In the absence of the choriocapillaris, localized macular hypoxia, oxidative stress, and inflammation upregulate VEGF, stimulating the growth of new blood vessels arising from the choroid (type 1 and 2 macular neovascularization [MNV]) or the retina (type 3 MNV) [65]. Nevertheless, the combination of immature blood vessel formation, vessel instability, and permeability (through loss of endothelial tight junctions pericytes), breakdown of the outer blood-retinal barrier, and inflammation are central to the pathogenesis of nAMD, suggesting a possible role for Ang-2 inhibition in its treatment [69, 70].

Preclinical studies have shown that Ang/Tie2 plays an important role in the choriocapillaris during its development and maintenance in the healthy adult eye, as well as in models of choroidal neovascularization (CNV) disease [53, 70, 71]. A high Ang-2/VEGF ratio promotes regression of neovascularization, whereas elevation of both Ang-2 and VEGF leads to increased neovascularization, suggesting that simultaneous inhibition of Ang-2 and VEGF could prevent further choroidal vasculature remodeling [45]. Findings in human tissue and isolated human cells have additionally highlighted the role of Ang/Tie2 in nAMD. In aqueous humor samples from patient eyes with nAMD, Ang-2 levels were elevated and correlated with disease severity [72]. It has also been observed in clinical trials of anti-VEGF monotherapy that when treatment leads to CNV regression in nAMD, patients may be left with macular atrophy and fibrosis at the site of regression; however, if type 1 MNV is appropriately treated, there may be protection from macular atrophy and fibrosis [73, 74]. Consequently, there is a need in nAMD for therapies that control and stabilize actively growing choroidal vasculature. In summary, similar to DME, findings from preclinical studies and from patients with nAMD suggest that Ang-2 could be a potential target for evaluation in this context due to its influence on vascular stability, independent from VEGF.

## Dual Ang-2 and VEGF inhibition in retinal vascular diseases

The synergistic relationship between Ang-2 and VEGF in the promotion of vascular leakage and inflammation supports the concept of dual pathway inhibition to promote vascular stability and improve outcomes beyond anti-VEGF therapies for patients with retinal vascular diseases. Studies in tumor models have suggested a compensatory relationship between Ang-2 and VEGF in pathologic angiogenesis, whereby inhibition of one molecule results in a shift to upregulate the other [75]. This may explain why long-term efficacy is difficult to maintain with VEGF inhibition alone, and provides further rationale for targeting Ang-2 and VEGF simultaneously [9, 14]. In a dual inducible mouse model in which both Ang-2 and VEGF could be overexpressed, Oshima et al. [39] showed that Ang-2 sensitized retina vessels to the angiogenic effects of VEGF. No data are available that show VEGF elevation can sensitize retinal vasculature to Ang-2 activity in a similar way. It is reasonable to expect that durable suppression of Ang-2 can limit the biologic activity of VEGF, reducing neovascularization and vessel permeability. This was demonstrated in transgenic mice with inducible Ang-2 expression during experimental hypoxia (i.e., when VEGF levels are high), when Ang-2 expression promoted retinal neovascularization [76]. Following resolution of hypoxia (i.e., when VEGF levels are low), Ang-2 expression promotes regression of retinal neovascularization. These findings support the notion that Ang-2 induces vascular stability after an angiogenic switch, in a VEGF-independent manner [26–28]. The study also showed that the context of VEGF elevation was important for the effect of Ang-2, as vasculature in the deep capillary bed was more sensitive to Ang-2 elevation during early embryonic development, with no effect on the superficial capillary bed [76]. At later stages of embryonic development, the morphology of the deep capillary bed was similar to that of wild-type mice.

In the eye, dual Ang-2/VEGF pathway inhibition in a mouse model of retinal angiomatous proliferation, using aflibercept in combination with an inhibitor (AKB-9778) of the negative regulator of Tie, VE-PTP, showed an additive effect on reducing subretinal neovascularization [53]. Similarly, in a mouse model of spontaneous CNV [77], dual VEGF-A/Ang-2 inhibition yielded reductions from baseline in CNV lesion leakage area versus either anti–VEGF-A or anti–Ang-2 monotherapy [77, 78]. Reductions in retinal inflammation adjacent to CNV lesions and photoreceptor apoptosis were also observed with dual VEGF-A/Ang-2 inhibition in this study. Dual VEGF/Ang-2 inhibition also decreased retinal leukocyte infiltration and aqueous humor inflammatory cells in a mouse model of endotoxin-induced uveitis, whereas Ang-2 or VEGF inhibition alone showed no effect [79].

Combination therapy with anti-VEGF and anti–Ang-2 agents has been evaluated in patients with retinal vascular disease. A phase 2 study in DME, RUBY, found that intravitreal aflibercept combined with the anti–Ang-2 antibody, nesvacumab, achieved similar BCVA gains to aflibercept monotherapy over 36 weeks; however, mean reductions in central subfield thickness (CST) and rates of CST resolution, as well as other anatomic parameters, were significantly higher with aflibercept/nesvacumab combination therapy [80]. ONYX, a phase 2 superiority study in nAMD, similarly showed that mean 36-week vision gains with aflibercept plus nesvacumab were not significantly different from those with aflibercept monotherapy, although trends toward greater mean CST reduction with combination therapy versus anti-VEGF monotherapy were observed [81]. Although the findings of RUBY and ONYX showed anatomic benefits with combined aflibercept and nesvacumab therapy, vision outcomes were comparable to aflibercept monotherapy; therefore, further development of this combination therapy for nAMD and DME was not supported [82]. Nevertheless, the results of these studies support the hypothesis that the addition of Ang-2 pathway inhibition may promote vascular stability and improve anatomic outcomes and durability beyond VEGF inhibition alone, based on the VEGF-independent activities of Ang-2.

## Faricimab: a novel bispecific antibody designed for intraocular use

The bispecific antibody faricimab (F. Hoffmann-La Roche Ltd., Basel, Switzerland) was developed on the premise that neutralization of both VEGF-A and Ang-2 may synergistically promote vascular stability in retinal vascular diseases, and improve outcomes for patients [77, 83]. Faricimab was designed using CrossMAb technology (F. Hoffmann-La Roche Ltd.) and is based on a human immunoglobulin G1 framework, with two fragment antigen-binding arms that bind VEGF-A and Ang-2 with high specificity and potency [77, 78, 83, 84]. The fragment crystallizable (Fc) region of faricimab is engineered to allow for faster systemic clearance, reduced systemic exposure, and reduced inflammatory potential. Removal of the Fc gamma receptor binding site has the potential to eradicate antibody-dependent cytotoxicity, antibody-dependent cell phagocytosis, and complement-dependent cytotoxicity. Deletion of the neonatal Fc receptor binding site prevents intracellular immunoglobulin G recycling, thereby reducing the systemic half-life of faricimab treatment compared with wild-type immunoglobulin G, and decreasing the likelihood of potential systemic toxicity [77]. In a laser-induced CNV model in non-human primates, intravitreal faricimab elicited a significantly greater reduction in CNV (as assessed by scored fluorescent angiograms) and vessel leakage compared with anti–VEGF-A and anti–Ang-2 monotherapy [77, 78].

In a phase 1 trial in patients with nAMD and subfoveal CNV refractory to prior anti-VEGF therapy (NCT01941082), faricimab (dosed from 0.5 to 6.0 mg) was well tolerated with an acceptable safety profile, and there was preliminary evidence of improved BCVA and reductions in CST (Table 1) [85].

**Table 1:** Summary of faricimab clinical studies in nAMD and DME

Completed studies				
Study name/identifier	Phase	Indication	Treatments	Findings
NCT01941082 [85]	1	nAMD and subfoveal CNV refractory to prior anti-VEGF	Faricimab, dose range 0.5–6.0 mg	Preliminary evidence of improved BCVA and CST Acceptable safety profile in dose range of 0.5–6.0 mg
AVENUE NCT02484690 [86]	2	Subfoveal CNV secondary to nAMD	Faricimab 1.5 mg Q4W; faricimab 6.0 mg Q4W; faricimab 6.0 mg Q8W after four initial Q4W doses; faricimab 6.0 mg after three initial ranibizumab 0.5 mg Q4W doses; ranibizumab 0.5 mg Q4W	Patients receiving faricimab achieved BCVA and anatomical improvements similar to ranibizumab over 36 weeks No new or unexpected safety signals
STAIRWAY NCT03038880 [87]	2	Subfoveal or juxtafoveal CNV secondary to nAMD	Ranibizumab 0.5 mg Q4W; Faricimab 6.0 mg four Q4W doses followed by Q12W or Q16W	Comparable improvements in BCVA, CST, and total lesion area between all three arms
TENAYA/LUCERNE NCT03823287/NCT03823300 [88–90]	3	nAMD with subfoveal CNV, or juxtafoveal or extrafoveal CNV with a subfoveal component	Aflibercept 2 mg Q4W for 3 doses followed by Q8W Faricimab 6.0 mg Q4W for 4 doses. Disease activity assessed at week 20 and 24; based on this, patients assigned to faricimab 6.0 mg Q8W, Q12W, or Q16W fixed dosing up to week 60, followed by T&E regimen after week 60*	Non-inferior BCVA gains for faricimab T&E vs aflibercept at year 1 Greater reductions in CST with faricimab T&E vs aflibercept at week 12; reductions comparable at year 1 45% of patients in faricimab 6.0 mg T&E arm on Q16W dosing Safety profile comparable to aflibercept Results maintained throughout year 2
BOULEVARD NCT 02699450 [91]	2	DME	Faricimab 6.0 mg Q4W, faricimab 1.5 mg Q4W, ranibizumab 0.3 mg Q4W	Visual acuity gains with faricimab 6.0 mg vs ranibizumab 0.3 mg in treatment-naïve patients Dose-dependent improvements in CST with faricimab in treatment-naïve and previously treated patients
YOSEMITE/RHINE NCT03622580/NCT03622593 [92–94]	3	DME	Aflibercept 2 mg Q8W after five initial Q4W doses up to week 16; faricimab 6.0 mg Q8W after six initial Q4W doses up to week 20; faricimab 6.0 mg T&E* after four initial Q4W loading doses up to week 12	Non-inferior BCVA gains with faricimab Q8W and T&E vs aflibercept at year 1 Greater CST reduction with faricimab Q8W and T&E vs aflibercept at year 1 Q12W or longer dosing in 72.4% of patients at week 52 on faricimab T&E Safety profile comparable to aflibercept Results maintained throughout year 2
**Ongoing studies**				
**Study name/identifier**	**Phase**	**Indication**	**Aim**	
AVONELLE-X NCT04777201 [95]	3	nAMD	Open-label extension study for long-term efficacy, safety, and durability of faricimab	
SALWEEN (ISRCTN69073386) [96]	4	nAMD (PCV)	Open-label study efficacy, durability, and safety of faricimab in PCV in Asia	
ALTIMETER NCT04597918 [97]	2b	DME	Exploratory open-label trial to investigate association between clinical endpoints, multimodal imaging assessments, and aqueous humor biomarkers in patients receiving faricimab	
RHONE-X NCT04432831 [98]	3	DME	Open-label extension study for long term efficacy, safety, and durability of faricimab	
ELEVATUM NCT05224102 [99]	4	DME	Open-label single arm study to improve understanding of faricimab in under-represented patient populations, and barriers that limit trial recruitment and retention in these populations	

^*^ Patients on T&E regimens could be extended up to Q16W

BCVA, best-corrected visual acuity; CNV, choroidal neovascularization; CST, central subfield thickness; DME, diabetic macular edema; nAMD, neovascular age-related macular degeneration; PCV, polypoidal choroidal vasculopathy; Q4W, every 4 weeks; Q8W, every 8 weeks; Q12W, every 12 weeks; Q16W, every 16 weeks; T&E, treat and extend

## Faricimab for the treatment of DME

BOULEVARD, a phase 2, randomized, controlled, 36-week study, evaluated the safety and efficacy of faricimab in 229 patients (both treatment-naïve and previously treated) aged ≥ 18 years with center-involved DME (BCVA of 73–24 Early Treatment of Diabetic Retinopathy study [ETDRS] letters; CST ≥ 325 µm). Patients received intravitreal faricimab 6.0 mg every 4 weeks (Q4W), faricimab 1.5 mg Q4W, or ranibizumab 0.3 mg (US Food and Drug Administration–approved DME dose) Q4W up to week 20 [91]. Faricimab showed statistically significant BCVA gains achieving superiority at week 24 from baseline with faricimab 6.0 mg dosed monthly, compared with monthly ranibizumab in treatment-naïve patients (*p* = 0.03). Anatomic outcomes were also improved with faricimab versus ranibizumab. No new or unexpected safety signals were reported (Table 1) [91].

The phase 3, multicenter, randomized, active comparator-controlled, double-masked, 100-week, non-inferiority trials YOSEMITE (NCT03622580) and RHINE (NCT03622593) were identically designed to assess the safety, efficacy, and durability of faricimab in anti-VEGF treatment-naïve and previously treated patients with DME (Table 1) [92, 93]. Patients aged ≥ 18 years with macular thickening secondary to DME involving the center of the fovea (CST ≥ 325 µm) and a BCVA of 25–73 ETDRS letters (20/320–20/40 approximate Snellen equivalent) were eligible for inclusion in the studies. In total, 1891 patients across 353 sites worldwide were randomized 1:1:1 to intravitreal faricimab 6.0 mg every 8 weeks (Q8W) after six initial Q4W doses; faricimab 6.0 mg according to a personalized treat-and-extend-based regimen (T&E) after four initial Q4W doses; or aflibercept consistent with its globally aligned posology, comprising five initial Q4W doses followed by Q8W injections through to week 96 [100, 101]. Patients in the faricimab T&E arm received faricimab 6.0 mg Q4W until they reached a CST < 325 µm, at or after week 12. Once achieved, treatment intervals were extended to Q8W, then could be maintained, extended by 4 weeks (up to every 16 weeks [Q16W]), or reduced by 4 or 8 weeks (to as low as Q4W) based on prespecified CST and BCVA criteria at active dosing visits. The T&E arm was designed to assess the durability of faricimab using a standardized method designed to replicate a T&E regimen as in routine clinical practice.

The primary endpoint of non-inferior 1-year vision gains with faricimab Q8W or T&E versus aflibercept Q8W was met in both YOSEMITE and RHINE. Adjusted mean BCVA change from baseline at the primary endpoint visits (averaged over weeks 48, 52, and 56) in the pooled DME population from YOSEMITE/RHINE was + 11.2 (95% confidence interval [CI], 10.5–12.0) ETDRS letters with faricimab Q8W, + 11.2 (95% CI, 10.4–11.9) with faricimab T&E, and + 10.5 (95% CI, 9.8–11.3) with aflibercept Q8W [102].

In YOSEMITE/RHINE, the faricimab arms achieved greater reductions in CST over 1 year of treatment compared with aflibercept [102]. Adjusted mean CST change from baseline at year 1 (averaged over weeks 48, 52, and 56; 95% CI) in the pooled DME population was –200.9 (–206.7 to –195.1) µm with faricimab Q8W, –192.4 (–198.1 to –186.6) µm with faricimab T&E, and –170.2 (–176.0 to –164.4) µm with aflibercept Q8W [102]. Greater reductions in CST with faricimab were also observed during the head-to-head phase, when all arms received the same number of doses (0–16 weeks) [102]. Through year 1, more faricimab-treated patients achieved absence of protocol-defined DME (CST < 325 µm) compared with aflibercept-treated patients (81%–89% with faricimab Q8W and 82%–85% with T&E versus 68%–74% with aflibercept Q8W, at weeks 48–56) [102]. In addition, more faricimab- versus aflibercept-treated patients achieved absence of intraretinal fluid through week 56 (41%–46% with faricimab Q8W and 33%–42% with T&E versus 22%–27% with aflibercept Q8W, at weeks 48–56) [102]. Rates of absence of subretinal fluid were ≥ 96% at week 52 and comparable across all three study arms [102]. The proportion of patients with rates of ≥ 2-step ETDRS Diabetic Retinopathy Severity Scale score improvement from baseline at week 52 was consistently > 40% across faricimab treatment arms (45.1% and 43.1% with faricimab Q8W and T&E, respectively), and was similar to those observed in the aflibercept Q8W arm (41.3%) [102]. Outcomes were achieved with 51.9% of patients in the faricimab T&E arms on Q16W dosing at the week 52 visit, and in 72.4% on every-12-week (Q12W) dosing or longer [102].

Overall, faricimab was well tolerated with an acceptable safety profile that was comparable to that of aflibercept [93]. Incidence of ocular events in the study eye was similar between patients receiving faricimab Q8W (37.3%), faricimab T&E (35.6%), or aflibercept Q8W (34.4%). Serious ocular events were also comparable between patients receiving faricimab Q8W (2.4%), faricimab T&E (3.0%), or aflibercept Q8W (1.3%). In both trials, rates of intraocular inflammation were low (1.3%, 1.4%, and 0.6% with faricimab Q8W, faricimab T&E, and aflibercept Q8W, respectively). Investigators reported no cases of retinal vasculitis or occlusive retinal vasculitis.

To summarize, the 1-year efficacy results of YOSEMITE and RHINE demonstrated that faricimab Q8W or T&E resulted in non-inferior vision gains versus aflibercept Q8W, while demonstrating improved disease control, through improved anatomic outcomes and the potential for extended durability of up to Q16W. Recently published results including year 2 of faricimab treatment from YOSEMITE and RHINE show that vision gains, anatomical control and treatment durability were maintained through the second year of treatment [94]. Together, these findings support the hypothesis of an anatomical benefit from Ang-2 inhibition in DME and suggest that dual Ang-2/VEGF-A pathway inhibition may promote vascular stability beyond that achieved with VEGF inhibition alone. Additional data are included in the published primary results of the YOSEMITE and RHINE trials [92–94].

## Faricimab for the treatment of nAMD

AVENUE was a phase 2, randomized, controlled, 36-week trial in 273 faricimab treatment-naïve patients aged ≥ 50 years with subfoveal CNV secondary to nAMD (BCVA 73–24 ETDRS letters [approximate Snellen equivalent 20/40–20/320]) (Table 1). It evaluated intravitreal faricimab 1.5 mg administered Q4W, faricimab 6.0 mg Q4W, faricimab 6.0 mg Q8W after four initial Q4W doses, faricimab 6.0 mg Q4W after three initial ranibizumab 0.5 mg Q4W doses, and ranibizumab 0.5 mg Q4W [86]. Patients who received either faricimab dose achieved and maintained BCVA and anatomic improvements, including CST, CNV area, and leakage, at a similar level to those achieved with ranibizumab [86]. A phase 2, randomized, controlled, 52-week trial, STAIRWAY, evaluated the efficacy and safety of extended dosing intervals with faricimab in 76 treatment-naïve patients aged ≥ 50 years with subfoveal or juxtafoveal CNV secondary to nAMD (BCVA and Snellen equivalents as in AVENUE) (Table 1). Intravitreal ranibizumab 0.5 mg administered Q4W was compared with faricimab 6.0 mg administered as four initial Q4W doses followed by dosing Q12W or Q16W through week 52 [87]. The BCVA gains at week 40 were comparable for faricimab Q12W or Q16W and ranibizumab, as were changes in CST and total lesion area [87]. Faricimab therefore demonstrated sustained efficacy at dosing intervals of up to Q16W. Faricimab was well tolerated, with an acceptable safety profile comparable to ranibizumab, and no new or unexpected safety events were identified in these phase 2 studies [86, 87].

The phase 3 TENAYA (NCT03823287) and LUCERNE (NCT03823300) trials were identically designed to evaluate the safety, efficacy, and durability of faricimab in treatment-naïve patients with nAMD (Table 1) [88, 89]. These were multicenter, randomized, active comparator-controlled, double-masked trials of 112 weeks’ duration. The trials included patients who were aged ≥ 50 years at enrollment, with a BCVA of 24–78 ETDRS letters (20/320–20/32 approximate Snellen equivalent), and either subfoveal CNV, or juxtafoveal or extrafoveal CNV with a subfoveal component. In total, 1329 patients with nAMD across 271 study sites worldwide were randomized 1:1 to aflibercept 2.0 mg Q8W after three initial Q4W doses, as per its globally aligned posology, or faricimab 6.0 mg [100, 101]. After four initial Q4W doses of faricimab, disease activity was determined based on CST and investigator-assessed BCVA and presence of macular hemorrhage. Patients with active disease at week 20 then received Q8W dosing through week 60, those with active disease at week 24 received Q12W dosing through week 60, and those with no active disease at weeks 20 and 24 were treated with faricimab at week 28 and remained on Q16W dosing through week 60. From week 60 to week 112, all patients in the faricimab arm were treated according to a T&E regimen, in which dosing intervals could be extended by 4 weeks, maintained, or reduced by 4 or 8 weeks from Q8W up to Q16W according to disease activity assessments.

The primary efficacy endpoint of non-inferiority in mean BCVA change from baseline at the primary endpoint visits (averaged over weeks 40, 44, and 48) with faricimab up to Q16W versus aflibercept Q8W was met in TENAYA and LUCERNE, with an adjusted mean gain from baseline of + 6.2 ETDRS letters (95% CI, 5.3–7.1) with faricimab and + 5.9 (95% CI, 5.0–6.7) with aflibercept in the pooled nAMD population [103]. At the primary endpoint visits, approximately 20% of patients gained ≥ 15 ETDRS letters from baseline with faricimab up to Q16W and with aflibercept Q8W, and 96% of patients across both treatment arms avoided losses of ≥ 15 ETDRS letters from baseline [104].

Anatomically, the adjusted mean changes in CST from baseline at the primary endpoint visits (averaged over weeks 40, 44, and 48) were comparable between faricimab up to Q16W and aflibercept Q8W, and were −137.0 µm (95% CI, −141.2 to −132.9 µm) and −130.1 µm (95% CI, −134.2 to −125.9), respectively [103]. However, greater reductions in CST with faricimab over aflibercept were observed during the head-to-head phase (0–12 weeks) [105]. These vision outcomes were achieved with approximately 45% of faricimab-treated patients in TENAYA/LUCERNE receiving Q16W dosing at week 48 [103].

In TENAYA and LUCERNE, faricimab was well tolerated and had an acceptable safety profile, with a low incidence of adverse events (AEs) leading to study treatment discontinuation [88]. Incidence of ocular AEs and serious ocular AEs through week 48 were generally similar between faricimab (ocular AEs, 38.3%; serious ocular AEs, 1.7%) and aflibercept (ocular AEs, 37.2%; serious ocular AEs, 2.0%). Rates of intraocular inflammation were low (2.0% for faricimab and 1.2% for aflibercept), and there were no investigator-reported events of retinal vasculitis or retinal occlusion associated with intraocular inflammation events in either study.

In summary, the 1-year efficacy results of TENAYA and LUCERNE demonstrated that faricimab up to Q16W offered non-inferior vision gains and improved anatomic outcomes versus aflibercept Q8W, supporting the hypothesis that dual Ang-2/VEGF-A pathway inhibition with faricimab may promote sustained efficacy for patients with nAMD. In TENAYA and LUCERNE, 1-year CST outcomes with faricimab were similar to those with aflibercept Q8W and were achieved with Q16W dosing in approximately 45% and with ≥ Q12W dosing in almost 80% of faricimab-treated patients. Year 2 results of faricimab treatment from TENAYA and LUCERNE show that vision gains, anatomical control, and treatment durability were maintained through the second year of treatment, with a greater proportion of patients achieving Q16W dosing versus year 1 [90]. Additional data are included in the published primary results of the TENAYA and LUCERNE trials [88–90].

## Discussion

### Clinical implications and conclusions

The introduction of anti-VEGF therapies revolutionized the management of retinal vascular disease. Real-world long-term outcomes with anti-VEGF monotherapy are improving, but they still fall below expectations. Possible approaches to improving VA and durability outcomes include increasing the dose, changing the drug delivery paradigm, and introducing a new mode of action. It is important to note that increased dosing of anti-VEGF agents has not led to improvements in clinical outcomes, particularly during the initial part of the study when dosing interval was matched with treatments [106–109]. Preclinical evidence suggests that dual Ang-2/VEGF inhibition may promote vascular stability and reduce the neovascularization and chronic inflammation compared with anti-VEGF inhibition alone [77–79].

Faricimab is a novel bispecific anti–Ang-2 and anti–VEGF-A antibody designed for intraocular use that has been evaluated in a phase 3 clinical trial program in retinal disease. In these studies, patients with DME or nAMD treated with faricimab demonstrated non-inferior vision gains, improved anatomical outcomes, and a similar safety profile versus aflibercept in the head-to-head phase, with ≥ 72% of patients in the faricimab T&E arms achieving Q12W dosing or longer at weeks 52 and 48 in YOSEMITE/RHINE and TENAYA/LUCERNE, respectively. The extended dosing intervals achieved with faricimab support the concept that Ang-2/VEGF-A–targeted therapies may address the unmet need for durable treatments that improve real-world outcomes and reduce the treatment burden compared with standard-of-care therapies for patients with retinal vascular diseases.

Results from studies assessing the correlation between anatomical outcomes and visual acuity are mixed. While some have shown that the absence of fluid is associated with visual improvements, others have demonstrated a poor correlation between the presence of fluid and visual outcomes, with vision loss not always associated with new fluid [110]. Further research is warranted to understand why the improvements in anatomical outcomes observed with faricimab in the phase 3 clinical trial program did not translate into improved vision outcomes versus aflibercept.

The promising results for patients with DME and nAMD suggest that faricimab may also be efficacious in other retinal vascular diseases. Phase 3 studies are under way in patients with macular edema secondary to central retinal or hemiretinal vein occlusion (COMINO; NCT04740931) [111], and in those with macular edema secondary to branch retinal vein occlusion (BALATON; NCT04740905) [112].

Results from the open-label extension study, RHONE-X (NCT04432831) (Table 1) [98], will inform the long-term safety, efficacy, and durability of faricimab in patients with DME. Similarly, the open-label extension study, AVONELLE-X (NCT04777201) (Table 1) [95], will provide further data on the safety, efficacy, and durability of faricimab in patients with nAMD. ELEVATUM (NCT05224102) (Table 1) is a phase 4 trial designed to improve understanding of faricimab in under-represented patients with DME, and the associated barriers that limit trial recruitment and retention in these populations [99]. SALWEEN (ISRCTN69073386) is a phase 4 study that will assess the efficacy, durability and safety of faricimab in polypoidal choroidal vasculopathy, a subtype of nAMD and a population under-represented in TENAYA/LUCERNE, in Asia (Table 1) [96]. Lastly, the phase 2b ALTIMETER (NCT04597918) (Table 1) biomarker hypothesis-generating study is exploring the associations between clinical endpoints, multimodal imaging assessments, and aqueous humor biomarker patterns in patients with DME treated with faricimab [97]. These studies may help demonstrate the benefits of Ang-2/VEGF-A co-inhibition in additional patient populations and over longer treatment periods.

Current evidence suggests that dual inhibition of Ang-2 and VEGF-A with faricimab may signal an important shift toward multitargeted treatment strategies for patients with DME, nAMD, and potentially other retinal vascular diseases, to improve outcomes versus anti-VEGF alone.
